# Buffalo Academy of Medicine

**Published:** 1900-06

**Authors:** J. C. Clemesha


### Society Proceedings.

### BUFFALO ACADEMY OF MEDICINE.

*Section on Medicine, April 11, 1900.*

REPORTED BY J. C. CLEMESHA, M. D., Secretary.

THE seventh regular meeting of the Medical Section, Buffalo Academy of Medicine, was held at the Academy parlors, Market Arcade, April 11, 1900. Meeting called to order 8.30 p. m. Dr. E. H. LONG in the chair. Members present, 30.

After the minutes of the last meeting were read and approved, Dr. MARCEL HARTWIG showed a child nine years of age suffering from a neurosis. The muscles of the back were atrophied or undeveloped, those of the lower limbs, especially the calf of the leg, abnormally hard. Discussion of this case was postponed.

Dr. DEWITT SHERMAN read a report of a case of post chicken-pox nephritis. A child, four years old, on January 24, 1899, became ill with follicular tonsilitis; on January 31, 1899, with chicken-pox. The eruption was not abundant and the symptoms mild. On February 8th, the urine was noted dark, blood-stained and scanty, with some slight tenesmus. Urinary analysis showed abundance of albumin, 2.10 of 1 per cent., blood corpuscles, epithelial and blood casts, kidney cells and leucocytes. The child gradually recovered. During the illness there was no edema, no symptoms of uremia. Ergot, alum and gallic acid were given to check hemorrhage. A milk diet, orange juice and mild purgatives were amongst other measures.

Dr. Sherman reviewed the literature and noted that very few cases had been reported, those chiefly by German observers.

Dr. H. R. HOPKINS then read a paper on The Treatment of Scarlatina, referring only to the hygienic conditions. For a number of years he has been in charge of the infectious wards of the Buffalo General Hospital and had arrived at some definite conclusions upon this subject, especially the lack of precision in the treatment of scarlatina. Introducing the subject of the contagiousness of typhoid fever and tuberculosis he stated that definite hygienic measures had been laid down as to the general line of treatment. In regard to scarlatina, lazy, halting, antiquated measures still prevailed. Scarlatina in its early stages is not infectious or contagious. By common consent the desquamation stage is the most active infectious state, but we do not know how active, and have at present no means of estimating the degree or period of infection; in fact, we have no single evidence that the desquamated epithelial cells have a particle of infective character. Of practical value is the method of sterilising the hands and feet of patients, so they may return home several days earlier. Dr. Hopkins has been using a paste of Dr. Robert Weir, who employed it for the surgical disinfection of his hands. It is a mixture of chlorinated lime and washing soda made into a paste. Nascent chlorine is liberated and exerts a powerful sterilising effect. Before using Weir's paste the average duration of stay in Kimberly Cottage was thirty-four days. Since the institution of sterilising methods, the average is now twenty-four days, and he believed it possible in the future, that when the fever has subsided the case will be closed and we shall not have to prolong the treatment to get rid of the infectiousness. Bacteriologists should furnish information as to the infectiousness of the desquamated particles and place in our hands a technique, so that a child may be treated in its own home with safety to others.

Dr. Irving M. Snow then read a paper on Normal Saline Solution in Gastro-enteric Diseases of Children. After an historical survey of the subject, commencing in 1884 when Gaetano first used saline solution in the treatment of cholera to the present, the essayist discussed the preparation, strength and modes of administration of this substance. During the past three years it has been employed in hospitals and private practice by him. In the hospital at Athol Springs patients generally arrive weakened and depressed and an injection of saline solution is generally given on their entrance with good effects. There are generally two classes of cases: (1) Those with high temperature, vomiting and diarrhea; an acute infection without intestinal lesion. (2) Those with moderate fever, intolerance of food, six stools daily; an infection with intestinal lesions. In a large number of cases the amelioration was temporary. In certain forms no good was noted, while in a smaller number the serum was life-saving. Syncope and acute crises might be tided over.

In conclusion, saline solution was (1) a prompt stimulant; (2) a replacer of fluid; (3) should be used only as an adjuvant.

DISCUSSION.

Dr. Charles Cary in discussing Dr. Hopkins's paper added a protest to some municipal methods in vogue which add to the unpopularity of reporting cases: (1) as in a family living over a store, the latter suffers; (2) house visitation after an attack, when much expense had to be endured by orders for new plumbing.

Dr. A. L. Benedict spoke on the several papers and asked Dr. Sherman if a bacteriological examination had been made on the child's throat at the time he was ill with tonsilitis. In discussing Dr. Snow's paper he gave preference to bowel washes over injections.

Drs. Frost, Jack, of Depew, B. G. Long, also added to the discussion.

The trustees asked an opinion from this section as to the choice of rooms and Dr. Hurd moved, seconded by Dr. Benedict, that the sense of the meeting was to seek other quarters, preferably those offered by the trustees of the Catholic Institute. Meeting adjourned at 10.50 p.m.

